# What Determines Spontaneous Physical Activity in Patients with Parkinson’s Disease?

**DOI:** 10.3390/jcm9051296

**Published:** 2020-05-01

**Authors:** Agnieszka Gorzkowska, Joanna Cholewa, Andrzej Małecki, Aleksandra Klimkowicz-Mrowiec, Jarosław Cholewa

**Affiliations:** 1 Department of Neurorehabilitation, Faculty of Medical Sciences in Katowice, Medical University of Silesia, 40-752 Katowice, Poland; agorzkowska@sum.edu.pl; 2 Institute of Physioterapy and Health Sciences, The Jerzy Kukuczka Academy of Physical Education, 40-065 Katowice, Poland; a.cholewa@awf.katowice.pl (J.C.); a.malecki@awf.katowice.pl (A.M.); 3 Department of Neurology, Jagiellonian University, Medical College, 31-503 Krakow, Poland; Aleksandra.Klimkowicz@mp.pl; 4 Department of Health Related Physical Activity and Tourism, The Jerzy Kukuczka Academy of Physical Education in Katowice, 40-065 Katowice, Poland

**Keywords:** Parkinson’s disease, physical activity, sedentary way, non-motor symptoms, apathy, dopaminergic therapy

## Abstract

Physical activity (PA) is a factor that may have an influence on the symptoms of Parkinson’s disease (PD). The aim of this study was to identify the potential determinants of spontaneous PA in a PD patient group. A total of 134 PD patients aged 65.2 ± 9.2 years with a Hoehn–Yahr scale score ≤4 and a Mini Mental State Examination (MMSE) score ≥24 were examined. For the study’s purposes, the authors analyzed age, sex, education, history of PD, dopaminergic treatment, the severity of PD symptoms using Unified Parkinson’s Disease Rating Scale (UPDRS), and Hoehn–Yahr scale. Additionally, all participants were evaluated through a set of scales for specific neuropsychiatric symptoms including depression, anxiety, apathy, fatigue, and sleep disorders. A linear regression analysis was used with backward elimination. In the total explanatory model, for 12% of the variability in activity (R^2^ = 0.125; F(16.133) = 2.185; *p* < 0.01), the significant predictor was starting therapy with the dopamine agonist (DA) (β= 0.420; t= 4.068; *p* = 0.000), which was associated with a longer duration of moderate PA. In the total explanatory model, for more than 13% of the variance in time spent sitting (R^2^ = 0.135; F(16.130) = 2.267; *p* < 0.01), the significant predictors were secondary education and the results of the UPDRS. The patients with secondary and vocational education, those starting treatment with DA and those with a less severe degree of Parkinson’s symptoms (UPDRS), spent less time sitting in a day. It is possible to identify determinants of spontaneous PA. It may elucidate consequences in terms of influence on modifiable conditions of PA and the proper approach to patients with unmodifiable PA factors.

## 1. Introduction

Parkinson’s disease (PD) is one of the most common neurodegenerative diseases and the most common form of parkinsonian syndrome. It affects 1% of people older than 60 years of age and 4% of people older than 80 years of age [1]. The main symptoms of PD primarily result from a dopaminergic deficit and include bradykinesia, tremors, rigidity, and postural instability, but in the course of PD, there are also numerous non-motor symptoms including neuropsychiatric, autonomic and gastrointestinal aspects [2].

The evidence collected thus far indicates that one of the important non-pharmacological interventions in the course of PD is the physical activity (PA) of the patients [3,4,5]. Even cortical activity tends to increase with PA like walking and balance tasks in older and PD groups compared to baseline conditions (sitting/standing) or controls [6]. The beneficial effects of PA on the central nervous system (CNS) are associated with many factors, including neurotrophic factors and, above all, the cerebral nerve growth factor and attributable neuroplasticity [7,8]. While these results are promising, current PD treatments are aimed at addressing motor symptoms, and there is no therapy focused on modifying the course of the disease [9].

So far, the literature has not elucidated the determinants of physical activity or inactivity in patients with PD. The present study analyzes the impact of sociodemographic factors, clinical features of the disease, and treatment on the time patients spend participating in spontaneous PA.

## 2. Material and Methods

### 2.1. Subjects

**Figure 1:**
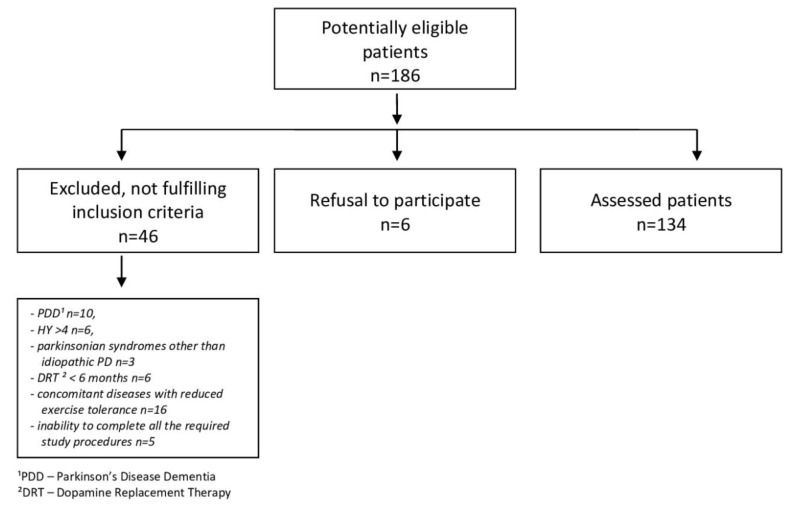
Flow-chart of patient selection.

A total of 134 PD patients aged 65.2 ± 9.2 years (61 women, 73 men), treated at the outpatient Neurology Clinic of the Silesian Medical University in Katowice, were examined. The study was approved by the Bioethics Committee of the Academy of Physical Education in Katowice. All participants signed the informed consent form. PD was diagnosed based on the principles of the United Kingdom Parkinson’s Disease Society Brain Bank. All other diseases in stable patients were under medical supervision. Figure 1 shows a flow chart of the participants’ recruitment.

In the examined group, the basic treatment was levodopa followed, in the frequency of use, by a DA (ropinirole or piribedil) The levodopa equivalent daily dose (LED) was calculated on the basis of the conversion ratios accepted based on the literature review [10]. The participants were patients with Hoehn–Yahr stage [11] ≤4 with a Mini-Mental State Examination (MMSE) score [12] ≥24 points and with a duration of symptoms and dopamine replacement therapy (DRT) ≥0.5 year. Characteristics of the respondents are presented in Table 1.

### 2.2. Methods

The study used a diagnostic survey with an authorial questionnaire for the purpose of gathering information on demographics, the clinical picture of PD, concomitant diseases, and treatment of the PD patient. The questionnaire was verified in terms of accuracy and reliability in the pilot studies preceding the main study.

The severity of PD symptoms was evaluated using the Unified Parkinson’s Disease Rating Scale (UPDRS) scale and the Hoehn–Yahr scale [11]. For mental performance, the MMSE was used [12]. PA was assessed using the International Physical Activity Questionnaire (IPAQ) [13] and the Minnesota Leisure Time Physical Activity Questionnaire (MLTPAQ) [14]. The questionnaires concerning activity were conducted by the researcher and covered a typical week of the subject’s life in March and November [15]. For the assessment of non-motor symptoms (NMS), the following evaluations were used: depression and anxiety—the Beck Depression Inventory (BDI) [16] and the Hospital Anxiety and Depression Scale (HADS) [17], apathy—the Apathy Scale (AS) [18], fatigue—the Parkinson’s Fatigue Scale (PFS−16) [19,20], sleep disturbances—the Pittsburgh Sleep Quality Ratio (PSQI) [21,22], and excessive daytime sleepiness—the Epworth Sleepiness Scale (ESS) [22].

For the analysis of PA, the data coming from IPAQ [13] and MLTPAQ [14] concerning the type of PA undertaken based on the level of difficulty (light, moderate, average), the duration of participation (hours/minutes), and the frequency during the week (number of days) were used.

To assess the results, the following operationalization of data concerning PA was conducted: The index of time of moderate weekly activity was calculated (ITMWA), which was established by the following formula: ITMWA = (average time of moderate physical activity from the MLTPAQ questionnaire + average time of moderate weekly activity from the IPAQ questionnaire)/2. On the basis of an empirical percentile division of the results obtained, the patients were divided into two groups: the 33% least active (ITMWA < 45 min a week) and the 33% most active (ITMWA > 2.5 h a week), which constituted the basis to characterize inactive and active patients.

Moderate weekly activity (ITMWA), which is calculated using the result of MLTPAQ and IPAQ, is an author’s concept developed for this work. In previous pilot studies, this parameter was verified by the method of competent judges used verified by Kendall test [23].

## 3. Results

The physically “inactive” PD patient group (PI-G), compared to the physically “active” PD patient group (PA-G), was characterized by a longer duration of the disease (8.2 ± 4.2 vs. 6.3 ± 4.0; *p* < 0.01) and less frequent treatment initiation with dopaminergic receptor agonist (DA) (1.9% (*n* = 1) vs. 21.8% (*n* = 12); *p* < 0.01). In addition, the PI-G had greater difficulties performing the activities of daily living (ADLs) assessed by UPDRS II (9.2 ± 5.5 vs. 7.0 ± 5.7; *p* < 0.05), and the PI-G more often suffered from apathy (15.9 ± 5.6 vs. 13.2 ± 5.4; 63.5% (*n* = 33) vs. 49.1% (*n* = 27); *p* < 0.05). The other analyzed factors did not reveal statistically significant differences between the PA-G and PI-G. The results of the assessment of all the factors listed using methodology tools are presented in Table 2.

In the context of the relatively small number of patients treated with piribedil (8 patients of the whole group) compared to ropinirole, separate analyses of the DA treatment in PI-G and PA-G group have been omitted.

To determine the relationship between subitems in the total score of each part of the UPDRS scale, the correlation of coefficients in both groups was calculated. In UPDRS part I, the largest correlation with total score was found for point 4 (Motivation/Initiative) in both groups, respectively (PA-G r = 0.65; PI-G r = 0.83); in part II, in PA-G with turning in bed r = 0.75, while in PI-G with walking r = 0.70. In part III of the UPDRS scale in PA-G, the total score showed the highest correlation with arising from a chair (r = 0.83), and in PI-G, with agility of the left leg (r = 0.78). In UPDRS part IV in PA-G—with symptoms anorexia, nausea or vomiting—r = 0.76, in PI-G, with the presence of “off” periods, r = 0.76, which was expected.

As potential factors impacting the duration of PA expressed by means of ITMWA, the following factors were taken into account: age, sex, education, type of medication initiating the therapy and the current treatment, LED, stage of disease based on the Hoehn–Yahr scale, severity of movement symptoms in part III of the UPDRS scale, degree of intensification of other Parkinson’s symptoms based on the UPDRS scale parts I + II + III, anxiety, depression, apathy, fatigue, and sleep disorders.

After analyzing the complete model, using an analysis of linear regression with the method of backward elimination for each explained variable, the optimum model of factors affecting PA was created to explain the largest variance of data. Only those models that explained more than 10% of variability are discussed below.

The complete model explained 12% of the variation in PA and was statistically significant (R^2^ = 0.125; F(16.133) = 2.185; *p* < 0.01). The only significant predictor was starting therapy with DA (β= 0.420; t= 4.068; *p* = 0.000), which was associated with a longer duration of moderate PA. While performing the analysis to determine the best predictors in accordance with the principle of step-wise approach regression, it was found that six predictors, with the two most significant being beginning the treatment with DA and the severity of the disease based on the Hoehn–Yahr scale (R^2^ = 0.171; F(6.133) = 5.585; *p* < 0.001), were responsible for 17% of the variability of ITMWA (Table 3).

Another linear regression analysis was carried out to determine the significance of individual factors in the time spent being sedentary during the day. The complete model with the same predictors as in the previous analysis, in which the variable to be explained was the time spent sitting, explained over 13% of the variance and was statistically significant (R^2^ = 0.135; F(16,130) = 2.267; *p* < 0.01). Significant predictors in this model were secondary education, aggravation of movement disorders in part III of the UPDRS scale and intensification of Parkinson’s symptoms in UPDRS scale, parts I + II + III. Assuming the best solution (i.e., the model with the highest R^2^) in accordance with the principle of step-wise approach regression, it was found that six predictors, the most significant of which were vocational and secondary education, commencement of treatment with DA and intensification of mobility symptoms in part III on the UPDRS scale (R^2^ = 0.193; F(7.130) = 5.429; *p* < 0.001), were responsible for over 19% of the variability of daily time spent sitting (in a sedentary way). Less time spent sitting during the day was observed in the patients with vocational and secondary education, whose first medication for the treatment of PD was DA and who had more intensified mobility symptoms (Table 4).

As all the models tested, except those presented above, explained less than 10% of the variance of dependent variables concerning PA, additional analyses of regression (taking into account the predictors that, in particular models, turned out to be significant) were conducted.

When using the ITMWA as a dependent variable in the model, the predictors were education, duration of the disease, starting treatment with DA, severity of the disease on the Hoehn–Yahr scale, and the level of depression symptoms (BDI). The analysis using the method of backward elimination showed that the optimal model takes into account only three predictors: duration of the disease, starting treatment with DA, and advancement of the disease on the Hoehn–Yahr scale. This model was significant and explained more than 15% of the variance (R^2^ = 0.151; F(3.133) = 8.85; *p* < 0.001). However, the only significant predictor turned out to be the start of treatment with DA, which was connected with increased duration of PA.

In the model where the variable was time spent sitting during the day, the predictors were education, intensification of mobility symptoms in part III of the UPDRS scale, intensification of parkinsonian symptoms based on the UPDRS scale parts I + II + III, commencement of DA treatment, the results of the PFS−16 fatigue scale, and excessive sleepiness (ESS). The analysis conducted with the method of reverse elimination excluded only the results from the PFS−16 scale, causing the obtained optimal model to be significant, which explained over 19% of the variance (R^2^ = 0.195; F(7.130) = 5.509; *p* < 0.001). It was found that the patients with secondary and vocational education, who started treatment with DA and those who had less intense parkinsonian symptoms (UPDRS scale parts I + II + III), spent less time in a sedentary way during the day (Table 5).

## 4. Discussion

The overall level of PA in the examined group of patients with PD was low, which is also indicated by the results of other authors [24]. In terms of NMS, the authors only noted lower apathy in the group of more physically active patients with PD. The other NMS had no influence on the level of PA. Moreover, many authors have reported a clear relationship between lower intensity of these symptoms and PA [25,26,27]. However, this activity was undertaken within a planned rehabilitation context, and it was not likely to be replaced with spontaneous PA as a control of NMS. The level of education turned out to be one of the most important factors affecting participating in PA more often and for a longer duration and shortening the time during the day spent sitting. Thus, in the model explaining over 15% of the variability in the number of days participating in moderate activity, vocational and higher education were the strongest predictors (*p* < 0.05). In previous research, the authors related a higher level of education with participating in health-oriented behaviors, including PA [28].

The results obtained in this study may indicate that patients with higher and secondary education have a greater awareness of pro-health behaviors and ways to create a healthy lifestyle. PA in this group may be a continuation of the previously practiced healthy behaviors, including various forms of PA. In addition, participating in PA in this group may also be a result of a better understanding of the nature of the disease and non-pharmacological measures to improve health conditions. However, patients with vocational education may treat PA as a continuation of work-related activity more often. Additionally, in the model explaining almost 20% of the variability in lack of time spent participating in PA during the day and secondary and vocational education were strong predictors (*p* < 0.05). Other authors also pointed to the importance of education in participating in PA in adults [29] and those diagnosed with PD [30].

One of the observations of the present study is that increased levels of PA were connected with the commencement of dopaminergic therapy with DA. In the model explaining nearly 20% of the variability in time spent sitting during the day, the start of dopaminergic therapy with DA was one of the four significant explanations (*p* = 0.030). It is possible that the group of patients starting treatment with DA have less initial motor dysfunction and experience motor problems that disrupt their activity to a lesser extent. This may not be associated only with the oligosymptomatic onset of the disease but also with its mild course. At the same time, it can be assumed that with the appropriate treatment, patients who report more severe parkinsonian symptoms with greater negative effects on daily functioning receive the levodopa drug first to initiate the treatment. LED did not affect the level of PA, which was also reported by other researchers [28]. It should also be taken into account that the reduced risk of motor complications in patients who use DA but not levodopa at the beginning of their treatment makes these patients more physically active. This topic requires further research. It has been reported that DA group drugs (described here as pramipexol) can result in physical exercise dependence, which is a form of impulse control disorder [31]. The authors also observed such disorders in the course of ropinirole therapy. However, it should be noted that this type of disorder may manifest in patients who are currently using the drug and will probably not affect patients who have discontinued DA therapy. A short assessment of the disorders of impulse control is necessary when excessive PA is recorded for a patient treated with DA. It is also one of the important reasons for the assessment of spontaneous PA in patients with PD, with the need to search for ways to best evaluate it and unify the methods of its examination.

Other strong predictors of the number of days with moderate PA in the model described above explained over 15% of the variance in this variable, except for education and commencement of treatment with DA, and included the continuation of treatment with DA in this group. This phenomenon seems to be connected with the analogous mechanisms related to starting therapy with DA. In turn, in the model explaining almost 20% of the variability in time spent sitting during the day, except for education and commencement of treatment with DA, the intensification of Parkinson symptoms assessed by the sum of the points from part I, II and III of the UPDRS scale was the strongest predictor in this case. Therefore, with reference to patients with PD, the time spent participating in PA becomes shorter for the benefit of time spent sitting. The result of the degree of intensification of Parkinson symptoms, both in terms of mobility and non-mobility, and the limitations in everyday functioning are generally related to increased disability in the middle-aged period, which has also been noted by other authors [32].

In the case of average weekly PA time, the model accounting for 17% of the variability included the following significant predictors: starting treatment with DA and lower stage of Hoehn–Yahr disease, which were associated with a longer duration of physical activity. In turn, among all significant factors that explained over 19% of the variance in time spent sitting during the day, the following were notable: vocational and secondary education; lesser total intensification of symptoms from parts I, II and III of the UPDRS scale and, as previously discussed, commencement of treatment with DA. All of the factors mentioned above were predicators of less time spent sitting during the day.

In this study, the importance of the Hoehn–Yahr scale for scientists was confirmed. The subsequent degrees of the scale reflect the symptoms determining patient mobility. The result of the Hoehn–Yahr scale also depends on the presence of posture disorders, which are usually connected with distinctive motor deterioration. The Hoehn–Yahr scale turned out to be more useful in the analysis of the movement aspects of the disease than Part III of the UPDRS, the paradoxically lower result of which is associated with a longer time spent sitting. This result may be related to the fact that with lower intensity of tremors or by slowing down, patients need less time to perform daily activities; hence, they can spend more time sitting, and this effect disappears when assessing the results of parts I and II of the UPDRS.

Despite the absence of differences between groups in the results for parts I, III and IV of UPDRS, it was found that in parts II, III and IV, other items for PA-G and PI-G correlated with the total score of these assessments. In addition, in part II, the total scores for PA-G and PI-G differed with statistical significance. The total result in PA-G had the highest correlation with night symptoms—turning in bed—while in PI-G, the highest correlation was found with gait disturbances, which can limit the activity of the subjects. However, in the case of PA-G in UPDRS part III, the highest correlation was shown by arising from a chair, a symptom that only applies to movement initiation, while in PI-G the highest correlation was found for disorders of the agility left leg, which is a symptom that persists constantly during activity. On the other hand, in the case of UPDRS part IV in PA-G, it was found that non-motor treatment complication—anorexia, nausea or vomiting—did not significantly affect motor activity, while in PI-G, a motor treatment complication—off periods predictable—was found to unequivocally impair the physical activity taken.

## 5. Conclusions

The results obtained in this study indicate the association between the selected clinical, demographic and therapeutic factors and the PA undertaken by PD patients. It may allow for better identification of the patients threatened by lack of activity and may help increase their activity levels.

## Figures and Tables

**Table 1:** Characteristics of the subjects.

Variable		M (SD)
**Sex: Women/Men (*n*)**		**61/73**
Age (years)		65.2 (9.2)
Place of residence (%)	A city with over 100 thousand inhabitants. A city with fewer than 100 thousand inhabitants.	60.5 39.5
Marital status (%)	Married Single	83.6 16.4
Education (%)	Basic Professional Medium High	7.5 20.9 41.0 30.6
Accompanying conditions (%)		84.3
The age of the start of symptoms (years) EOPD/MOPD/LOPD (%)		57.9 (11.1) 21.6/66.4/12.7
The duration of the disease (years)		7.3 (4.2)
Hoehn–Yahr scale (degrees)		2 (0.6)
Time to start treatment (years)		1.4 (1.5)
Daily levodopa equivalent dose—LED (mg)		755.4 (418.7)
Levodopa/dopaminergic agonist (%)		96/52

EOPD, early onset Parkinson’s disease; MOPD, middle age onset Parkinson’s disease; LOPD, late onset Parkinson’s disease.

**Table 2:** Characteristics and results of the physically active and physically inactive subjects.

Variable	PA-G (*n* = 55)	PI-G (*n* = 53)	*p* Value
Age (years)	66.3 ± 8.1	65.5 ± 10.0	0.516 ^1^
Sex (W—women, M—Men)	W—45.5% (*n* = 25 M—54.5% (*n* = 30)	W—47.2% (*n* = 25) M—52.8% (*n* = 28)	0.858 ^2^
The duration of the disease (years)	6.3 ± 4.0	8.2 ± 4.2	*p* < 0.01 ^1^
Age of onset of (years)	65.4 ±8.1	66.3 ±10.0	0.622 ^1^
UPDRS I (points)	1.3 ± 1.9	1.6 ± 1.4	0.405 ^1^
UPDRS II (points)	7.0 ± 5.7	9.2 ± 5.5	*p* < 0.05 ^1^
UPDRS III (points)	24.9 ± 12.5	27.1 ± 13.0	0.246 ^1^
UPDRS IV (points)	1.8 ± 1.7	2.3 ± 2.8	0.381 ^1^
Hoehn–Yahr scale (degree)	2.2 ± 0.6	2.4 ± 0.6	0.055 ^1^
Dyskinesia (%)	20.0 (*n* = 11)	24.5 (*n* = 13)	0.218
Motor fluctuation (%)	38.2 (*n* = 21)	45.3 (*n* = 24)	0.946
Treatment: levodopa/DA (%)	96.4 (*n* = 53)	98.1 (*n* = 52)	0.580 ^2^
Start of treatment with levodopa (%)	74.5 (*n* = 41)	83.0 (*n* = 44)	0.282 ^2^
Start of treatment with DA (%)	21.8 (*n* = 12)	1.9 (*n* = 1)	*p* < 0.01 ^2^
Time to initiate treatment (years)	1.3 ± 1.4	1.0 ± 1.5	0.212 ^1^
LED (mg)	730.4 ± 433.4	797.6 ± 414.7	0.177 ^1^
Current Levodopa dose (mg)	674.5 ± 410.9	746.1 ± 387.3	0.710
Depression BDI (M ± SD) (% with depression)	9.5 ± 6.4 23.6 (*n* = 13)	11.9 ± 8.6 35.8 (*n* = 19)	0.287 ^1^
Depression HADS (M ± SD) (% with depression)	4.9 ± 3.7 20.0 (*n* = 11)	5.3 ± 3.7 26.4 (*n* = 14)	0.462 ^1^
Anxiety HADS (M ± SD) (% with anxiety)	5.7 ± 3.9 29.1 (*n* = 16)	5.1 ± 3.4 24.5 (*n* = 13)	0.517 ^1^
Apathy AS (M ± SD) (% with apathy)	13.2 ± 5.4 49.1 (*n* = 27)	15.9 ± 5.6 63.5 (*n* = 33)	*p* < 0.05 ^1^
Fatigue PFS−16 (M ± SD) (% with fatigue)	2.8 ±0.9 27.3 (*n* = 15)	3.1 ±1.0 45.3 (*n* = 24)	0.078 ^1^
Sleep disorders PSQI (M ± SD) (% with sleep disorders)	6.4 ± 3.3 56.4 (*n* = 31)	6.2 ± 3.6 52.8 (*n* = 28)	0.634 ^1^
Excessive daytime sleepiness ESS (M ± SD) (% with excessive daytime sleepiness)	6.3 ± 4.9 21.8 (*n* = 12)	7.7 ± 5.3 39.6 (*n* = 21)	0.167 ^1^

PA-G—Physically Active Patients, PI-G—Physically Inactive Patients, ^1^ Mann–Whitney U test; ^2^ chi-squared test.

**Table 3:** Analysis of backward regression, which determines % of the variability of ITMWA, conditioned by the group of socio-demographic and clinical predictors in the group of patients with Parkinson’s disease.

Depended Variable	Model	Predictors	R^2^	F	*p*	ϐ	t	*p*
ITMWA	Complete model	Sex	R^2^ = 0.125; F(16,133) = 2.185; *p* < 0.01			0.007	0.083	0.934
		Age				0.041	0.452	0.652
		Professional education				0.141	0.944	0.347
		Medium education				0.092	0.551	0.582
		High education				0.206	1.266	0.208
		Levodopa treatment				0.138	1.493	0.138
		DA treatment				0.033	0.364	0.717
		MAOBI treatment				−0.047	−0.547	0.585
		Amantadine treatment				0.010	0.119	0.905
		Anticholinergic treatment				0.004	0.044	0.965
		Start of treatment with levodopa				0.157	1.483	0.141
		Start of treatment with DA				0.420	4.068	0.000
		LED				−0.087	−0.892	0.374
		Hoehn–Yahr scale				−0.210	−1.821	0.071
		UPDRS part III				0.352	1.181	0.240
		UPDRS part I + II + III				−0.242	−0.785	0.434
	Optimal model highest value R^2^	Higher Education	R^2^ = 0.171; F(6.133) = 5.585; *p* < 0.001			0.105	1.261	0.210
		Levodopa treatment				0.120	1.421	0.158
		Start of treatment with Levodopa				0.153	1.537	0.127
		Start of treatment with DA				0.440	4.595	0.000
		Hoehn–Yahr scale				−0.238	−2.283	0.024
		UPDRS part III				0.144	1.400	0.164

R^2^—amount of explained variance, F—value of Fisher’s statistics, *p*—statistical significance, ϐ—significance of the predictor in the model, t—result of Student’s *t*-test.

**Table 4:** Analysis of backward regression, which determines % of variability of time spent in the sedentary position conditioned by the group of socio-demographic and clinical predictors in the group of patients with Parkinson’s disease.

Depended Variable	Model	Predictors	R^2^	F	*p*	ϐ	t	*p*
Time spent in the sedentary position	Complete model	Sex	R^2^ = 0.135; F(16.130) = 2.267; *p* < 0.01			−0.013	−0.149	0.882
		Age				0.070	0.768	0.444
		Professional education				−0.262	−1.787	0.077
		Medium education				−0.339	−2.026	0.045
		High education				−0.198	−1.218	0.226
		Levodopa treatment				0.017	0.178	0.859
		DA treatment				0.007	0.077	0.939
		MAOBI treatment				0.126	1.461	0.147
		Amantadine treatment				0.032	0.376	0.708
		Anticholinergic treatment				−0.002	−0.025	0.980
		Start of treatment with levodopa				0.007	0.062	0.951
		Start of treatment with DA				−0.151	−1.457	0.148
		LED				−0.029	−0.290	0.772
		Hoehn–Yahr scale				−0.006	−0.052	0.958
		UPDRS part III				−0.817	−2.705	0.008
		UPDRS part. I + II + III				1.047	3.362	0.001
	Optimal model highest value R^2^	Professional education	R^2^ = 0.171; F(6.133) = 5.585; *p* < 0.001			−0.289	−2.124	0.036
		Medium education				−0.362	−2.320	0.022
		Higher education				−0.213	−1.388	0.168
		Treatment MAOBI				0.125	1.541	0.126
		Start of treatment with DA				−0.162	−1.996	0.048
		UPDRS part III				−0.813	−2.849	0.005

R^2^—amount of explained variance, F—value of Fisher’s statistics, *p*—statistical significance, ϐ—significance of the predictor in the model, t—result of Student’s *t*-test.

**Table 5:** Analysis of backward regression, which determines % of the variability of ITMWA and time spent in the sedentary position in a day in the group of patients with Parkinson’s disease (solely optimum models with the highest R^2^ are presented).

Optimal Model	Predictors	R^2^	F	*p*	ϐ	t	*p*
ITMWA	Duration of the disease	R^2^ = 0.151; F(3.133) = 8.85; *p* < 0.001			−0.114	−1.379	0.170
	Start of treatment with DA				0.348	4.306	0.000
	Hoehn–Yahr scale				−0.105	−1.273	0.205
Time spent in the sedentary position	Medium education	R^2^ = 0.195; F(7.130) = 5.509; *p* < 0.001			−0.375	−2.407	0.018
	Professional education				−0.294	−2.172	0.032
	Higher education				−0.202	−1.322	0.188
	UPDRS part. III				−0.589	−1.939	0.055
	UPDRS parts I + II + III				0.787	2.535	0.013
	Start of treatment with DA				−0.177	−2.190	0.030
	ESS				0.147	1.677	0.096

R^2^—amount of explained variance, F—value of Fisher’s statistics, *p*—statistical significance, ϐ—significance of the predictor in the model, t—result of Student’s *t*-test.
